# Synthesis, Physicochemical Characterization using a Facile Validated HPLC Quantitation Analysis Method of 4-Chloro-phenylcarbamoyl-methyl Ciprofloxacin and Its Biological Investigations

**DOI:** 10.3390/ijms241914818

**Published:** 2023-10-01

**Authors:** Mostafa F. Al-Hakkani, Nourhan Ahmed, Alaa A. Abbas, Mohammad H. A. Hassan, Hossameldin A. Aziz, Ali M. Elshamsy, Hazim O. Khalifa, Mohamed A. Abdelshakour, Mohammed S. Saddik, Mahmoud M. A. Elsayed, Marwa A. Sabet, Mohamed A. El-Mokhtar, Mosa Alsehli, M. S. Amin, Ahmed M. Abu-Dief, Hamada H. H. Mohammed

**Affiliations:** 1 Department of Research, Development, and Stability, UP Pharma, Industrial Zone, Arab El Awamer, Abnoub 76, Assiut 71745, Egypt; nourhanahmedsultan23071993@gmail.com (N.A.); alaa.a.abbas93@gmail.com (A.A.A.); 2 Department of Medical Laboratory Technology, Higher Technological Institute for Applied Health Sciences in Minya, Minya 71511, Egypt; moh.hassan1512@science.au.edu.eg; 3 Department of Pharmaceutical Chemistry, Faculty of Pharmacy, New Valley University, New Valley 72511, Egypt; hossamaziz85@pha.nvu.edu.eg; 4 Department of Pharmaceutical Chemistry, Faculty of Pharmacy, Deraya University, Mina, New Minia 61768, Egypt; ali.elshamsy@deraya.edu.eg; 5 Department of Veterinary Medicine, College of Agriculture and Veterinary Medicine, United Arab Emirates University, Al Ain P.O. Box 1555, United Arab Emirates; hazimkhalifa@uaeu.ac.ae; 6 Department of Pharmacology, Faculty of Veterinary Medicine, Kafr Elsheikh University, Kafr El Sheikh 33516, Egypt; 7 Department of Pharmaceutical Analytical Chemistry, Faculty of Pharmacy, Sohag University, Sohag 82524, Egypt; mohamed.abdelshakour@pharm.sohag.edu.eg; 8 Department of Pharmaceutics and Clinical Pharmacy, Faculty of Pharmacy, Sohag University, Sohag 82524, Egypt; mohammed.sherif@pharm.sohag.edu.eg (M.S.S.); mahmoudalmenshawy@pharm.sohag.edu.eg (M.M.A.E.); 9 Department of Microbiology and Immunology, Faculty of Pharmacy, Sphinx University, New-Assiut 71684, Egypt; marwasabet@gmail.com; 10 Department of Medical Microbiology and Immunology, Faculty of Medicine, Assiut University, Assiut 71515, Egypt; elmokhtarma@aun.edu.eg; 11 Department of Chemistry, College of Science, Taibah University, Madinah P.O. Box 344, Saudi Arabia; mhsehli@taibahu.edu.sa (M.A.); meahassan@taibahu.edu.sa (M.S.A.); 12 Chemistry Department, Faculty of science, Ain Shams University, Cairo 11566, Egypt; 13 Department of Chemistry, Faculty of Science, Sohag University, Sohag 82524, Egypt; 14 Department of Pharmaceutical Chemistry, Faculty of Pharmacy, Sohag University, Sohag 82524, Egypt; hamada.hashem@pharm.sohag.edu.eg

**Keywords:** ciprofloxacin, HPLC, validation, antibacterial, anticancer, DNA gyrase, docking studies

## Abstract

A novel derivative of ciprofloxacin (Cpx) was synthesized and characterized using various analytical techniques, including FT-IR spectroscopy, UV-Vis spectroscopy, TEM and SEM analysis, ^1^H NMR, ^13^C NMR, and HPLC analysis. The newly prepared Cpx derivative (Cpx-Drv) exhibited significantly enhanced antibacterial properties compared to Cpx itself. In particular, Cpx-Drv demonstrated a 51% increase in antibacterial activity against *S. aureus* and a 30% improvement against *B. subtilis*. It displayed potent inhibitory effects on topoisomerases II (DNA gyrase and topoisomerase IV) as potential molecular targets, with IC50 values of 6.754 and 1.913 µg/mL, respectively, in contrast to Cpx, which had IC50 values of 2.125 and 0.821 µg/mL, respectively. Docking studies further supported these findings, showing that Cpx-Drv exhibited stronger binding interactions with the gyrase enzyme (PDB ID: 2XCT) compared to the parent Cpx, with binding affinities of −10.3349 and −7.7506 kcal/mole, respectively.

## 1. Introduction

Recently, the world has experienced rapid advancement and competition in the pharmaceutical industry. This is evident within the context of the evolving situation surrounding the coronavirus and diverse cancer ailments. Faced with this worldwide crisis, the resilience and harmonious collaboration among diverse scientific disciplines in their pursuit of identifying a secure solution were prominently demonstrated [1,2]. Antibiotic resistance has emerged as one of the most significant global health issues of the 21st century. Antibiotic resistance is reaching dangerously high levels worldwide, especially in developing countries [3].

**Figure 1:**
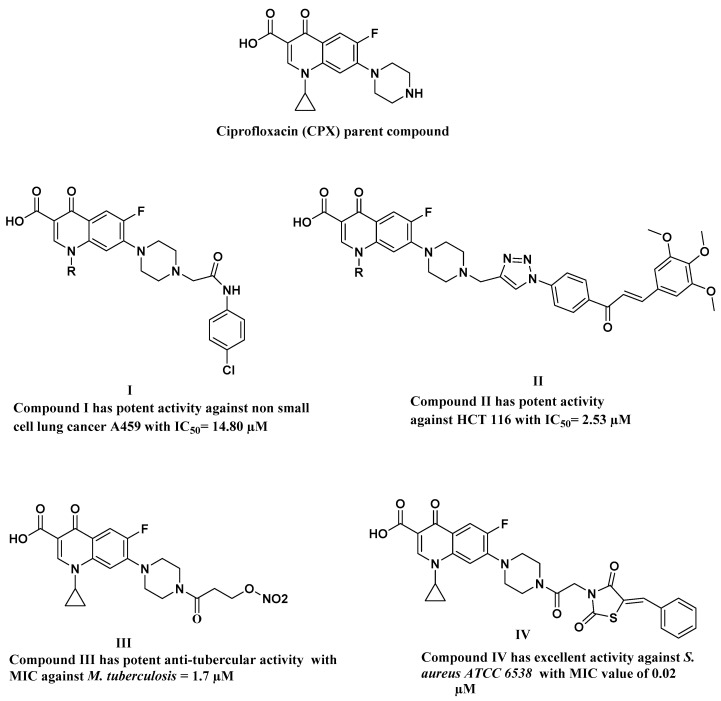
Chemical structure of Cpx and its derivatives; compounds **I** and **II** with potent anticancer activity, compounds **III** and **IV** with potent antimicrobial activities.

Therefore, there is an urgent need to discover new, more appropriate antibiotics in light of the mutations of pathogenic and deadly microorganisms. Antibiotics are essential for contemporary human patients because they cure and prevent diseases. The use of pharmaceuticals has grown throughout history [4]. Among the most famous families of antibiotics common in modern times are the family of macrolides, beta-lactams, cephalosporins, and fluoroquinolones. Fluoroquinolones are synthetic broad-spectrum bactericidal agents that include the vast majority of antibiotics being used in various treatment approaches, such as ciprofloxacin (Cpx), garenoxacin, gatifloxacin, gemifloxacin, levofloxacin, and moxifloxacin [2]. Fluoroquinolones act by inhibiting the activities of DNA topoisomerase II, resulting in the accumulation of double-stranded DNA breaks and bacterial cell death. In addition, fluoroquinolones have various biological activities including antitubercular, antifungal [5], anti-inflammatory [6], anti-Alzheimer’s [7], antiviral [8], anti-malarial [9], and antitumor activities [10,11]. Cpx is one of the most important examples of the second-generation fluoroquinolones and the fifth most widely used generic antibiotic in the world due to its broad antibacterial activity [12] against both Gram-positive and Gram-negative bacteria and because it can also be used in wound dressing [13,14,15,16,17,18]. The importance of Cpx is also due to its application in managing prevalent conditions such as respiratory tract infections, urinary tract infections, prostatitis, cellulitis, malignant otitis externa, chancroid, anthrax, endocarditis, and gastroenteritis [19]. Cpx’s full chemical name, according to IUPAC, is 1-cyclopropyl-6-fluoro-1,4-dihydro-4-oxo-7-(1piperazinyl)-3-quinoline carboxylic acid (Figure 1), and it has a molecular mass of 331.35 g/mol and empirical formula C_17_H_18_FN_3_O_3_ [20]. 

Several types of research have indicated that *N*-4 piperazinyl substitution of quinolones’ nucleus reduces zwitterion characteristics, improves physiochemical properties, and enhances antibacterial activity towards Gram-positive strains, and increases lipophilicity, which is considered one of the most important factors taken into consideration for the design of new anticancer agents. 

Based on the above-mentioned aspects, various derivatives have been synthesized, aiming to increase antibacterial activity or shift biological activity towards anticancer activity [9], as presented in Figure 1.

From this point, scientists started thinking about finding more derivatives of Cpx to achieve the desired results in terms of their superiority over its basic counterpart. Recently, Hamada et al. synthesized several derivatives of Cpx as listed in their published paper [21], which reported the preparation of seven derivatives to improve the weak anticancer activity of Cpx. Among these derivatives is compound **I** (Figure 1), which has a mass of 498.94 g/mol and the empirical formula C_25_H_24_FN_4_ClO_4_. 

The efficacy of Cpx-Drv (Figure 1, compound **I**) has been evaluated and found to manifest a very good efficacy in this regard as an anticancer agent [21] against fifteen cell lines. However, Hamada and co-authors did not examine the anti-microbiological activity of Cpx-Drv against different species of Gram-negative and Gram-positive bacteria. They also did not provide a direct analysis method to detect and determine Cpx-Drv in a quick way that expresses the assay of this derivative and compares it with the main drug, the parent from which it is derived, i.e., Cpx.

Therefore, the purpose of this work is to prepare Cpx-Drv using the same approach as previously reported [21], but with more physicochemical characterizations. A UV-RP-HPLC analysis method with full validation procedures was implemented. Moreover, anti-microbiological activity against different bacterial species was demonstrated.

## 2. Results and Discussion

### 2.1. FTIR Analysis

**Figure 2:**
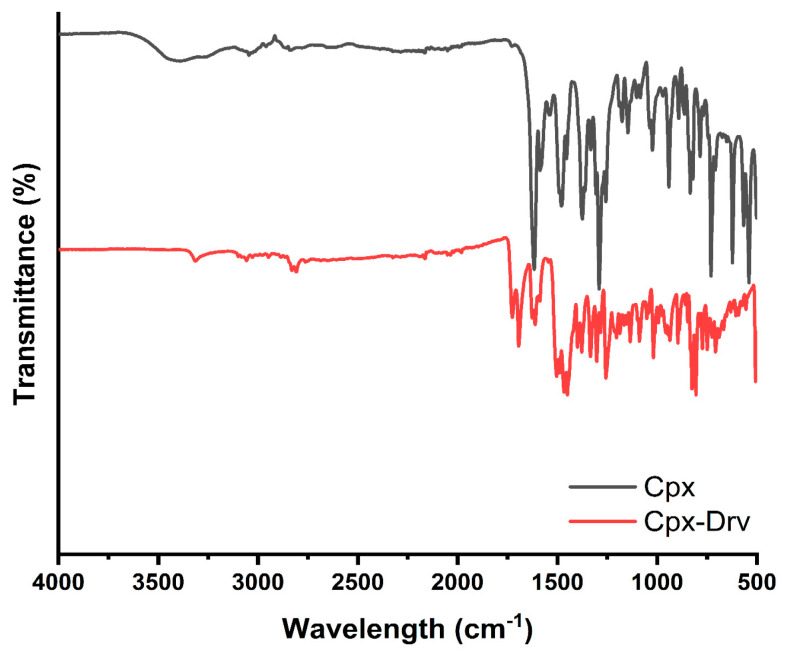
FTIR spectra of Cpx and Cpx-Drv.

The FTIR spectra of the as-prepared Cpx and Cpx-Drv are recorded in the mid-infrared region in the range of (500–4000 cm^−1^). The frequency of the bands for the different functional groups could be used to identify the molecule’s structure. Figure 2 and Table 1 provide a rapid summary for the identification of the functional groups of Cpx and Cpx-Drv. Table 1 shows some small deviations in the band positions, which indicate that the majority of peaks are shared between the original molecule Cpx and Cpx-Drv. The changes in the frequencies are attributed to the difference among the intermolecular distances according to the change in the dipole moment of the molecule [1]. 

### 2.2. UV-Vis Spectroscopy Identification

**Figure 3:**
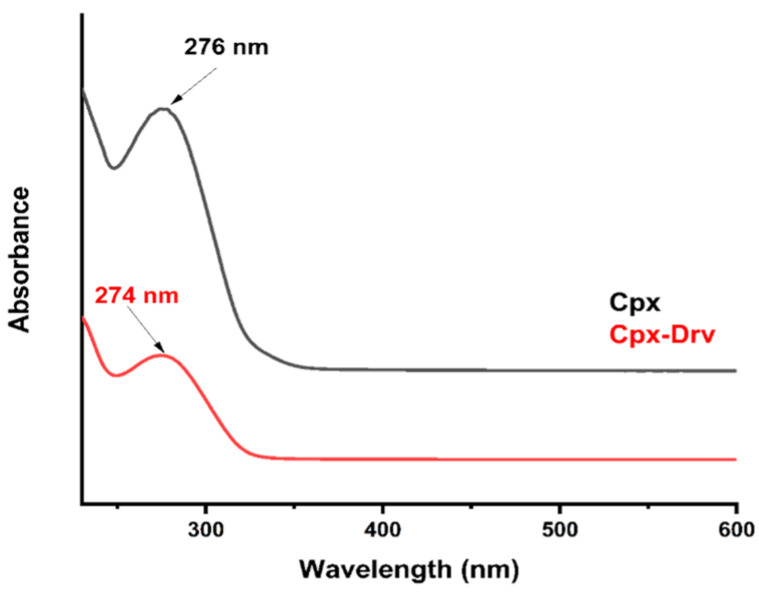
UV-Vis spectra scan of Cpx and Cpx-Drv.

Individual scan analysis of Cpx and Cpx-Drv samples was conducted in the range of 230–600 nm. Figure 3 shows the hypsochromic shift as a shorter wavelength in the maximum spectral band position was achieved from 276 nm to 274 nm. This change could be attributed to the higher molecular mass, including the structure relationship change according to Figure 1, compound **I**, where higher absorption is illustrated for Cpx-Drv. 

### 2.3. TEM and SEM Analysis

**Figure 4:**
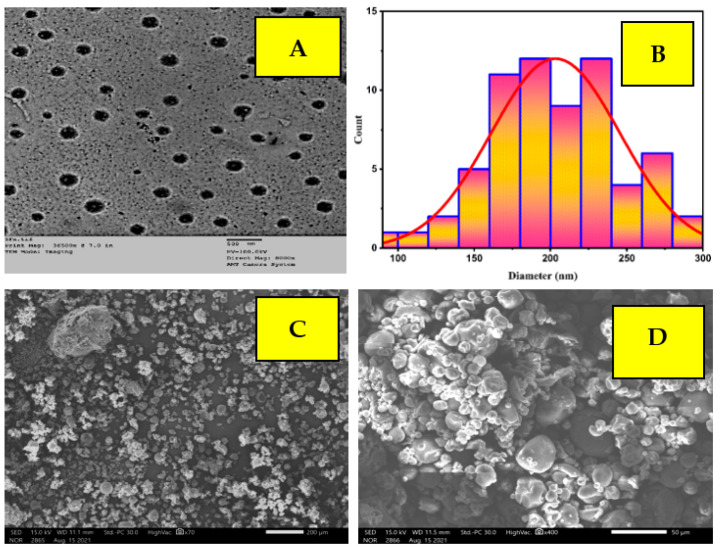
Cpx-Drv: (**A**) TEM image; (**B**) particle size distribution; (**C**,**D**) SEM images.

TEM and SEM analyses of Cpx-Drv are shown in Figure 4. The nanostructure integrity of the created Cpx-Drv was proven by TEM and SEM imaging on particles with a size range of 97.9–286.8 nm; the average particle size was found to be 203.3 nm and the median was 200.9 nm with a standard deviation of 42.3 nm. The monodispersed spherical shape was investigated in the absence of any agglomeration. Due to its monodispersibility and small particle size, the as-prepared Cpx-Drv can penetrate micro-organism cells as an antimicrobial, antiviral, or anticancer agent [22]. Moreover, due to these features, Cpx-Drv has the ability to become suspended in solution to form a suspension for oral administration of a drug.

### 2.4. ^1^H NMR and ^13^C NMR for Cpx-Drv “1-Cyclopropyl-6-fluoro-4-oxo-7-(4-(2-oxo-2-(4-chlorophenyl)amino)ethyl) piperazin-1-yl)-1,4-dihydroquinoline-3carboxylic Acid”

**Figure 5:**
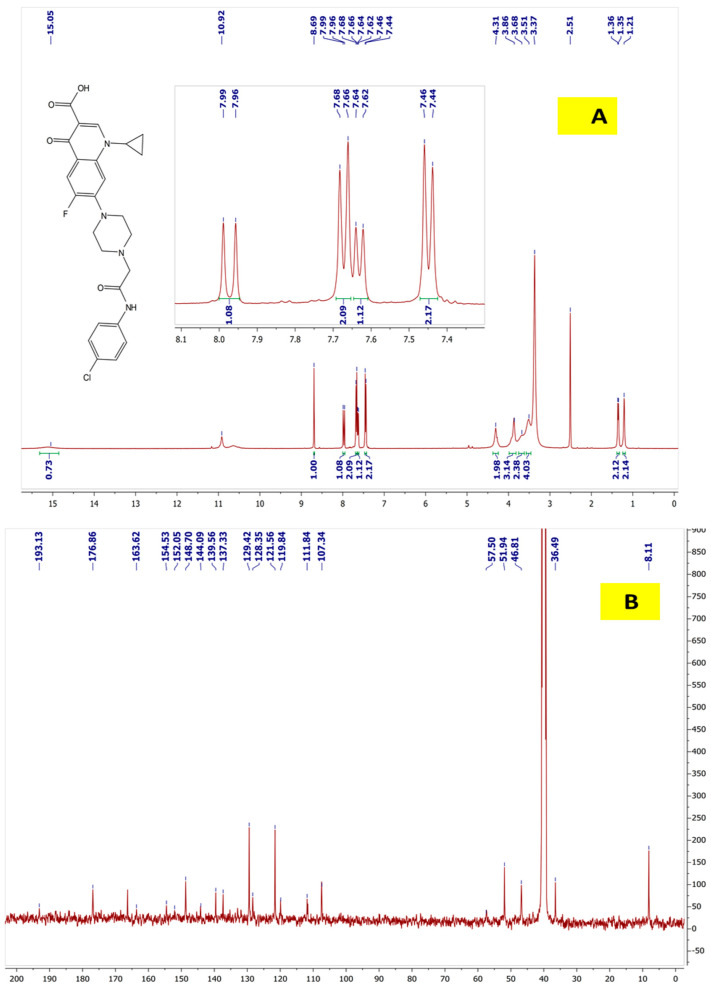
(**A**) ^1^H NMR and (**B**) ^13^C NMR for Cpx-Drv.

Pale yellow crystal; (0.90 g, 88.30% yield); mp 237–239 °C; (Figure 5) manifested the ^1^H-NMR (400 MHz, DMSO-d6) δ 1.19–1.22 (2H, m, cyclopropyl-H), 1.34–1.37 (2H, m, cyclopropyl-H), 3.50–3.55 (4H, m, piperazinyl-H), 3.65–3.67 (2H, m, piperazinyl-H), 3.85–3.89 (3H, m, piperazinyl-H and cyclopropyl-H), 4.31 (2H, s, -N-CH2-CO), 7.45 (2H, d, J = 8 Hz, Ar-H), 7.63 (1H, d, J = 8 Hz, H-8), 7.67 (2H, d, J = 8 Hz, Ar-H), 7.98 (1H, d, J = 12 Hz, H-5), 8.69 (1H, s, H-2), 10.92 (1H, s, -NH-CO) 15.05 (1H, brs, COOH); ^13^C-NMR (100 MHz, DMSO-d6) δ 8.11, 36.49, 46.81, 51.94, 57.50, 107.34, 111.84, 119.84, 121.56, 128.35, 129.42, 137.33, 139.56, 144.09, 148.70, 152.05, 154,53, 163.62, 176.86, 193.13.

### 2.5. Water Content Determination

The moisture or water content can be quantified using a semi-micro technique, allowing for the measurement of water content, which can be represented as either moisture or crystalline water, expressed as a percentage. This method finds widespread application, particularly in the analysis of water content in pharmaceuticals and dietary supplements, whether supplied as solids or liquids [3]. The Karl Fischer method (KFT) was employed to measure the water content and was found to be 8.13%. 

### 2.6. HPLC Quantitative, Qualitative Analysis, and Method Validation Check

**Figure 6:**
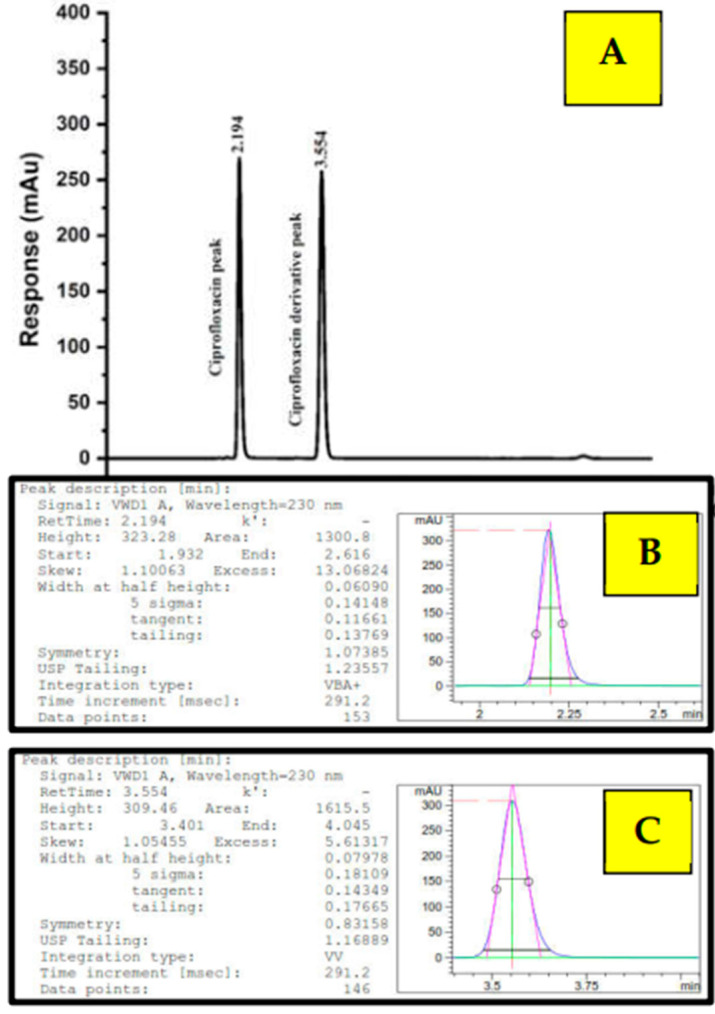
(**A**) HPLC chromatogram of Cpx and Cpx-Drv peaks at retention times of 2.194 min and 3.554 min, respectively; (**B**) USP tailing of Cpx; (**C**) USP tailing of Cpx-Drv.

According to the molecular structure as depicted in Figure 1, the elution of Cpx had a lower retention time, whereas Cpx-Drv has a higher molecular mass, so it was eluted at the end. In RP-HPLC, the basic rule is “likes dissolve likes or likes attract likes”. Because Cpx-Drv is more hydrophobic than Cpx, Cpx-Drv was eluted after Cpx, and this is the reason to use a high ratio of organic solvent. Cpx and Cpx-Drv peaks appeared at about 2.2 and 3.5 min, respectively, as seen in Figure 6. Under the optimized parameters of the analytical method, these ranged from 2.1 to 2.4 and 3.4 to 3.9 minutes for Cpx and Cpx-Drv peaks, respectively, over all the parameter changes. Table 2 and Table 3 show the high performance of the intended analysis method where the peak area RSD % ≤ 2.0%, USP tailing ≤ 2.0, theoretical plates ≥ 2000, and resolution ≥ 1.5 [23,24,25]. Hence, based on the obtained system suitability testing data, the method showed excellent validity through a wide range of retention times. 

The results manifested excellent linearity, with R^2^ = 0.99999 for both Cpx and Cpx-Drv peak concentrations in the range of 5–200 µg/mL against their intensity responses. Moreover, LOD and LOQ limits were estimated, as shown in Table 4, where it was found that the current method could be used for both of the components, especially Cpx-Drv to determine low levels. The accuracy findings of the studied range (35–60 µg/mL) from the targeted concentration of 100% = 50 µg/mL for each of Cpx and Cpx-Drv were found to be within the acceptance criteria (98–102%), as displayed in Table 4 [26,27]. Moreover, there were no undesirable effects as a result of the presence of the two components, in addition to a high recovery percentage.

The RSD% of peak areas was employed to assess the repeatability of the analyte using six different preparations at the same desired concentration (50 µg/mL of Cpx and Cpx-Drv), as shown in Table 5. The RSD% for the intra-precision and the inter-precision measures were found to be within the repeatability criteria requirements of ≤2.0%. Moreover, the method was robust, rugged, selective, and specific according to the output data, as manifested in Table 5, where the major two peaks were found to be separated with a highly satisfactory and reasonable resolution of ≥1.5. The forced degradation not only reveals any further degradation or new peaks for both Cpx and Cpx-Drv, but also represents strong evidence for drug stability under aggressive degradation conditions.

### 2.7. Antimicrobial Activity

**Figure 7:**
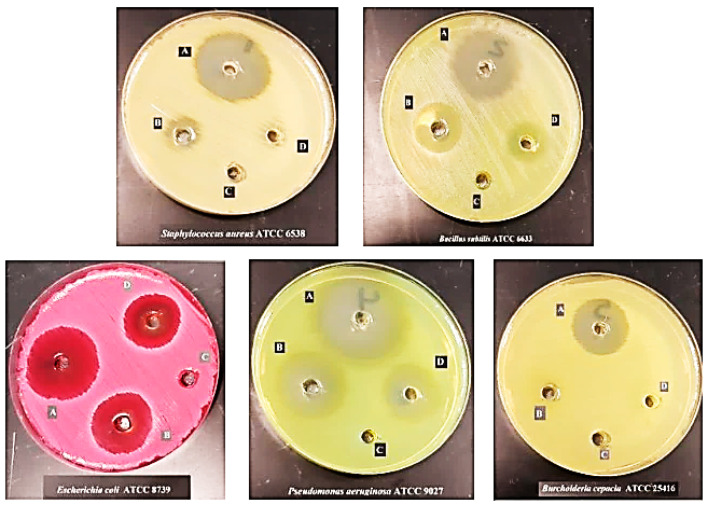
Different concentrations of Cpx-Drv: (A) 100 μg/mL; (B) 50 μg/mL; (C) 25 μg/mL; (D) Cpx control against different bacterial standard strains.

The antimicrobial activity of Cpx-Drv against Gram-positive, Gram-negative, and yeast standard strains is shown in Figure 7. The results revealed that there was a notable outperformance in the antibacterial activity for Cpx-Drv in comparison with Cpx. This was obvious from measuring the inhibition zone around each concentration against the tested strains. The high activity of Cpx-Drv against Gram-positive bacteria in comparison to Gram-negative bacteria was noted. The antibacterial activity of Cpx-Drv was found to be higher than that of Cpx by 51-fold against *S. aureus* and 30-fold against *B. subtilis* for Gram-positive bacteria.

Regarding Gram-negative bacteria, Cpx-Drv exhibited the greatest effect against *B. cepacia*. The fold increase reached 100%, whereas the standard Cpx did not show any antimicrobial activity against the tested strain of *Burkholderia* sp. In the case of the *E. coli* strain, the fold increase reached 26.3% and increased to 35.5% for *P. aeruginosa* compared to Cpx only. On the other hand, Cpx-Drv used in this study did not show any antifungal activity for each of Cpx-Drv or Cpx, as shown in Table 5. This enhancement of antibacterial activity is most likely due to the bacterial aggregation of Cpx-Drv, which increased the exposure of bacteria cells to Cpx-Drv and consequently increased the concentration of Cpx-Drv inside the cell. The increase in the inhibition zone diameter indicates enhanced antibacterial activity, as shown in Table 6.

### 2.8. DNA Gyrase and Topoisomerase IV Inhibition Assays

**Figure 8:**
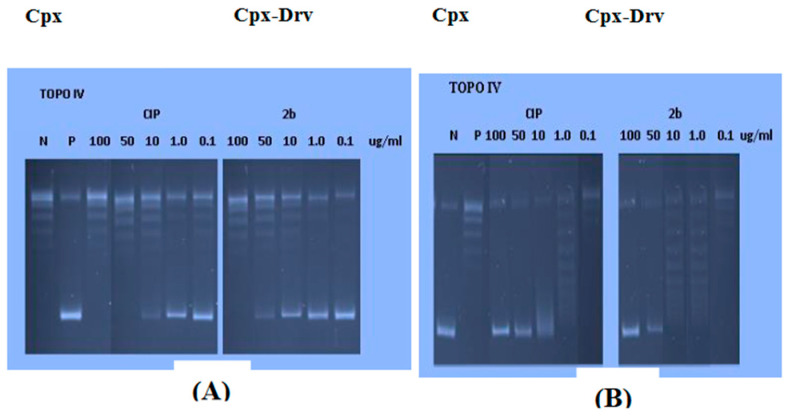
(**A**) DNA gyrase inhibitory assay; (**B**) DNA topoisomerase IV inhibitory activity.

Fluoroquinolones block DNA topoisomerase II enzymes (DNA gyrase and topoisomerase IV), as shown in Figure 8, which transiently cause DNA double-strand breaks as they negatively supercoil DNA. Fluoroquinolones inhibit DNA strands from passing through breaks and halt DNA replication, which causes cell death, by stabilizing the covalent enzyme-DNA adducts known as the cleaved complex. As a result, strong evidence for the inhibition of DNA topoisomerases can be found in the ability of the target compound, Cpx-Drv, to create a cleaved complex starting from the supercoiled plasmid. Using Cpx as a reference drug, agarose gel electrophoresis was used to assess the target compound’s ability to produce cleaved complexes starting with supercoiled pBR322. Ethidium bromide, a DNA intercalating chemical, was utilized in gel experiments to evaluate the stimulation of DNA breakage and inhibition of DNA supercoil relaxation. It is simple to distinguish relaxed DNA from nicked and linear species when ethidium bromide is added because closed circular DNA species positively supercoil when it is present. The results demonstrated that Cpx-Drv exhibits significant activity against DNA gyrase and topoisomerase IV, with IC50 values of 6.754 and 1.913 g/mL, but is less potent than Cpx. This is due to the role of *N*-4-piperazinyl substitution of Cpx in the enhancement of antibacterial activities by improving physiochemical parameters, decreasing zwitterions, and increasing lipophilicity. The improvement in physiochemical parameters has greater effect on antibacterial activity than the decrease in topoisomerase II inhibition of compound Cpx-Drv.

Cpx and Cpx-Drv were tested for their capacity to prevent DNA breakage by topoisomerase II enzymes. Results showed that Cpx-Drv is less powerful than Cpx against DNA gyrase and topoisomerase IV, with IC50 values of 6.754 and 1.913 g/mL, respectively. This suggests that the novel Cpx-Drv may have an additional antibacterial mechanism [28,29] (Table 7). 

### 2.9. Docking Studies

**Figure 9:**
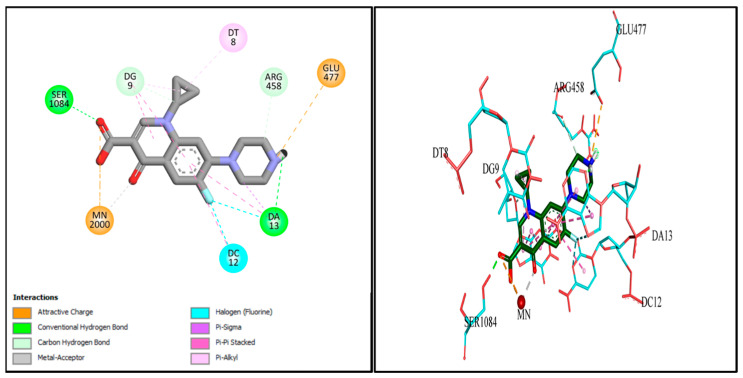
2D- and 3D-binding modes of Cpx within the gyrase-active site.

**Figure 10:**
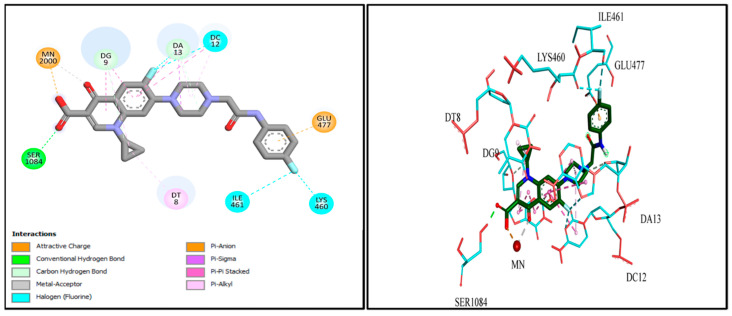
2D- and 3D-binding modes of Cpx-Drv within the gyrase-active site.

Molecular docking investigations were conducted to explore the potential interactions of Cpx-Drv in the active site of the *S. aureus* gyrase enzyme (PDB: 2XCT). Redocking of the co-crystallized Cpx revealed several distinct interactions with the target enzyme’s active site, including chelation with Mn^2+^, hydrophobic interactions, hydrogen bonding, and pi-cationic interactions with DNA nucleotide bases (Figure 9). Furthermore, Cpx-Drv demonstrated its ability to interact with the gyrase-active site through hydrophobic interactions and additional hydrogen bonding with DNA nucleotide bases and amino acid residues (Figure 10). These findings align with the observation that Cpx-Drv exhibits stronger antibacterial activity compared to its parent compound Cpx. A summary of all possible binding interactions between Cpx and Cpx-Drv is provided in Table 8. Notably, the binding affinity of Cpx-Drv to the gyrase-active site was superior to that of Cpx, with respective values of −10.3349 and −7.7506 kcal/mol.

### 2.10. Anticancer Activity

**Figure 11:**
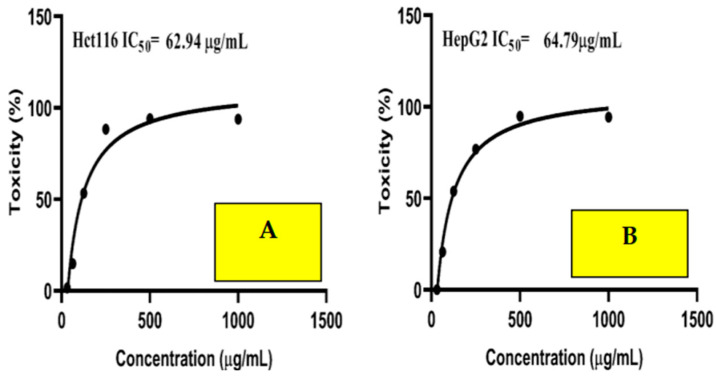
Cytotoxicity of Cpx-Drv against (**A**) HCT116 and (**B**) HepG2 cell lines.

The highly concentration-sensitive nature of Cpx-Drv, even at low concentrations, is revealed in Figure 11. Furthermore, the cytotoxicity of cells was found to be 93.9% and 93.3% at a dose of 1000 µg/mL in the HCT116 and HepG2 cell lines, respectively. The IC50 was estimated and found to be 62.94 and 64.79 µg/mL, respectively. The results revealed that Cpx-Drv has significant cytotoxic activity against HCT116 and HepG2. This result confirmed the previous finding of anticancer activity for the as-prepared Cpx-Drv [21]. Moreover, another reason for the anticancer activity of Cpx-Drv may be its particle size, which was found to be 203.3 nm, as revealed by TEM analysis. This small particle size was believed to facilitate the penetration into the cell line to destroy its biosystem and prevent tumor cells from reproducing. The anticancer mechanisms of Cpx-Drv were investigated and results showed that it exhibited four different mechanisms, including inhibition of MDR1, human topoisomerase I, and human topoisomerase II, as well as enhancement of the effects of paclitaxel (PTX) on microtubule assembly. 

## 3. Materials and Methods

### 3.1. Materials

Cpx material was provided as a complimentary sample by UP Pharma (Assuit, Egypt). Acetonitrile HPLC-grade (ACN), potassium dihydrogen phosphate, HCl 37%, and NaOH (Scharlau, Spain) were used. Deionized water was filtered through a 0.45 μm nylon membrane filter prior to use. 

### 3.2. Synthesis of the Target Compound Cpx-Drv

Cpx-Drv. was prepared as previously reported by Hamada et al. in their approach to new derivatives of Cpx [21] as follows. A mixture of the N-acyl-4-chloroaniline (2.2 mmol) in acetonitrile (10 mL), ciprofloxacin hydrochloride (2 mmol), and TEA (0.404 g, 4 mmol) was heated under reflux for 12-18 h. The formed precipitate was filtered off while hot, washed with acetonitrile, and dried under vacuum to produce the target compound Cpx-Drv.

### 3.3. Characterizations

FTIR was conducted using a KBr disc method using a Thermo Fisher Nicolet iS10 FTIR spectrometer with a wavenumber range of 4000–500 cm^−1^. UV-Vis absorption measurements were carried out in the range of 230–600 nm utilizing a PerkinElmer (LAMBDA 40 UV/Vis) spectrophotometer with a quartz cell having a 1 cm path length at room temperature. Morphological examination of Cpx-Drv was performed by scanning electron microscopy (SEM; JSM IT 200) and transmission electron microscopy (TEM; JEOL JEM-100C XII)). 

Cpx and Cpx-Drv assay analyses were implemented using the HPLC model HP 1100 series.

Melting points were determined using a Stuart’s electrothermal melting point instrument and were uncorrected. NMR spectra (400 MHz for 1H, 100 MHz for 13C) were observed using a DMSO-d6 on Bruker AM400 spectrometer with tetramethyl silane as the internal standard. Chemical shift (d) results are provided in parts per million using DMSO-d6 as solvent, and coupling constants are designated as (J) in Hz. Splitting patterns are designated as follows: s, singlet; d, doublet; dd, doublet of doublet; t, triplet; q, quartet; m, multiplet; br s, broad singlet.

The semi-micro determination of water was performed using *KFT* (701-703 KF titrinio). A reaction as oxidation/reduction of the Karl Fischer reagent was used for estimation of the water percentage. The reagent was composed of sulfur dioxide, iodine, and resin that includes a nitrogen atom with at least one unshared electron pair free from pyridine [30]. The as-synthesized Cpx-Drv mass of about 100 mg was presented to the *KFT* in the presence of methanol as a reaction medium. The following equations were used for determination of water percentage:

Titer (mg/mL) = (mg of added water standard)/ (mL of *KFT*) (1)

Water (%) = (Titer × mL of the *KFT* for Cpx-Drv × 100)/ (Cpx-Drv mass) (2)

### 3.4. HPLC Quantitative, Qualitative Analysis and Method Validation Check

The HPLC method was developed and validated on the basis of the International Conference on Harmonization (ICH) validation guidelines [26,27]. Several trials were conducted to reach the optimum parameters for better separation and detection of the two peaks of Cpx and Cpx-Drv in one chromatogram. The trials to obtain Cpx and Cpx-Drv started together in one chromatogram.

#### 3.4.1. Chromatographic System Configuration

Cpx and Cpx-Drv assay determinations were performed using the HPLC model HP 1100 series with variable wavelengths. The separation was achieved using a RP C18 BDS column (250 mm × 4.6 mm × 5 μm) (Thermo Scientific). The mobile phase consisted of 0.07 M of KH_2_PO_4_ in deionized water: ACN in a ratio (3:7, *v/v*) at a flow rate of 1.2 mL/min, with UV detection at 230 nm at room temperature and injection volume 20 μL.

#### 3.4.2. System Suitability Check

First, both Cpx and Cpx-Drv were injected individually to determine the dedicated retention time. A system suitability test was conducted via analysis of six replicates of the same sample solution prepared by dissolving an amount of Cpx and Cpx-Drv to obtain a solution concentration of 50 µg/mL using mobile phase as a solvent.

#### 3.4.3. Linearity and Range

The linearity test was conducted using different seven concentrations ranging from 10% to 400% of Cpx and Cpx-Drv. The working concentrations were prepared from 5–200 µg/mL. Every solution was injected twice.

#### 3.4.4. LOD and LOQ

LOD and LOQ were estimated from the linearity calibration curve using the following equations:

LOD = 3.3 σ/S (3)

LOQ = 10 σ/S (4)

where σ is the standard error of concentration and area response and S is the slope of the linearity calibration curve.

#### 3.4.5. Accuracy and Recovery

Both are two sides of the same coin and are used interchangeably. Accuracy describes the degree of closeness of the measured concentration value to the claimed theoretical concentration [23,24]. Accuracy was determined by weighing three different weights for each of Cpx and Cpx-Drv to produce theoretical concentrations at 35 µg/mL, 50 µg/mL, and 60 µg/mL. The linearity equation was used to determine the actual concentration, then the accuracy % was calculated using the following equation:

Accuracy %= Actual Conc.%/Theoretical Conc.% × 100 (5)

The accuracy acceptance limit for the assay method lay within 98.0–102% of the claimed concentration.

#### 3.4.6. Precision (Repeatability and Intermediate Precision)

Repeatability was demonstrated using six different individual determinations of the desired concentration of the intended method (100% = 50 µg/mL) of Cpx and Cpx-Drv, which were performed using the same instrument on the same day by the same analyst [27,30,31,32]. The repeatability acceptance limit for the assay method should not be greater than 2.0%, which is expressed as RSD% of the rear response or assay content [27].

Other aspects to the intermediate precision may appear and be assessed as robustness and ruggedness. If the analytical method resists the slight changes that are deliberately applied to it and shows approximately the same separation efficiency, the method is called robust. These changes include changing the flow rate, the organic solvent ratio, the buffer ratio, and the temperature. Contrary to the definition of robustness, the analysis method is defined to be rugged if it manifests the results with the same efficiency when the remarkable changes are applied that are believed to affect the desired result. Ruggedness was assessed via a change in the HPLC column, analysis on another instrument, analysis by another analyst, or analysis on different days. Both robustness and ruggedness were assessed at Cpx and Cpx-Drv concentrations of 50 µg/mL for each, which were performed as system suitability and repeatability tests.

#### 3.4.7. Specificity and Selectivity

Accelerated degradation was assessed by utilizing acid hydrolysis as 0.1 M of HCl, base hydrolysis as 0.1 M of NaOH, and light degradation over 6 h to reveal the stability-indicating features. The main reason for this test is that the analysis method can distinguish and separate between the principle intended components of Cpx and Cpx-Drv that we are looking for among the others that co-eluted with them. The acceptance criteria for the assay method’s specificity and selectivity are that the resolution value should be greater than 1.5 [26].

### 3.5. Antimicrobial Activity

The prepared antibiotic Cpx-Drv was subjected to evaluation of its antimicrobial activity against some reference standard bacterial and fungal strains, including 3 species of Gram-negative bacteria: *Escherichia coli* ATCC 8739 (*E. coli*), *Burkholderia cepacia* ATCC 25416 (*B. cepacia*), and *Pseudomonas aeruginosa* ATCC 9027 (*P. aeruginosa*). Two species of Gram-positive bacteria, *Staphylococcus aureus* ATCC 6538 (*S. aureus*) and *Bacillus subtilis* ATCC 6633 (*B. subtilis*), were also included. Furthermore, the yeast of *Candida albicans* ATCC 10231 (*C. albicans*) was also included [33,34,35,36]. The antimicrobial activity against the selected standard strain was evaluated and compared to the activity of Cpx. An overnight incubated microbial culture was suspended and the turbidity was adjusted to the equivalent 0.5 McFarland standard (1.5 × 10^8^ cfu/mL) [4,37,38].

#### 3.5.1. Agar Well Diffusion Method

The antimicrobial activity of Cpx-Drv and Cpx against the tested standard strain was determined using the agar well diffusion method. Briefly, Cpx-Drv was dissolved in sterile distilled water and serial dilution was undertaken to obtain various concentrations (25, 50, and 100 μg/mL) used for testing their antimicrobial activities [17]. 

#### 3.5.2. Antimicrobial Activity Bioassay

The prepared inoculums of standard strains (1 mL) were spread using a glass spreader to ensure an even distribution of the inoculums in Muller Hinton agar as a general medium, Cetrimide agar for *Pseudomonas* sp., MacConkey agar for *E. Coli*, and Sabouraud Dextrose agar for yeast. Wells were made by punching into the agar surface with a sterile cork borer. Using a micropipette, 150 L from each concentration was separately added to a single well. For bacterial strains, the inoculation plates were incubated at 37 °C for 24 h, and at 28 °C for 48 h for the yeast strain. The antimicrobial activity was estimated by measuring the diameter of the inhibition zone surrounding the wells. Cpx reference standard antibiotic was used as a positive control. Finally, the minimum inhibitory concentration (MIC) was defined as the lowest concentration that inhibits the growth of each strain [39,40,41]. All tests were carried out in triplicate.

### 3.6. Topoisomerase II Inhibition Assays

#### 3.6.1. *S. aureus* Gyrase Supercoiling Assay

In accordance with the prescribed procedures derived from the literature, *S. aureus* DNA gyrase assay was carried out [42]. In three separate replicate runs, the novel chemical Cpx-Drv was dissolved in DMSO, serially diluted at doses of 100, 10, 1, and 0.1 M, and then tested in reaction mixtures. A total solution volume of 30 L was used containing 40 mM HEPES, 10 mM magnesium acetate, 500 mM potassium glutamate, 2 mM ATP, 0.05 mg/mL albumin, and Relaxed pBR322. At 37 °C, the DNA gyrase from *S. aureus* was incubated for 30 to 60 min. Staph. aureus gyrase DNA gyrase supercoiling reactions were stopped by adding 30 L of STEB and 30 L of chloroform/isoamyl alcohol (v:v, 24:1); the result was then centrifuged for one minute, and 20 L of this was loaded on a 1% agarose gel and run at 75V for roughly two hours. The gel was stained using ethidium bromide in water (0.5 mg/L). The fluorescent images were recorded using a UV transilluminator imaging device at a wavelength of 300 nm. The fluorescence intensity of the supercoiled plasmid reaction result was quantitated using the Imag Quant program (Molecular Dynamics). By applying nonlinear regression analysis in Graph Pad Prism, the results as IC50 values (concentration of the tested substance that results in 50% inhibition of enzyme activity) were calculated [42].

#### 3.6.2. *S. aureus* Topoisomerase IV Decatenation Assay

IC_50_ values for compound Cpx-Drv and Cpx for Topo IV decatenation were determined. The following substances were utilized in this test: Topo IV Assay Buffer (provided as 5X), 50 mM Tris-HCl (pH 7.5), 5 mM magnesium chloride, 350 mM potassium glutamate, 5 mM DTT, and 1.5 m MATP, which were stored at or below −200 C. Quantities of 50 mM Tris-HCl (pH 7.5), 1 mM EDTA, 1 mM DTT, and 40% (*v/v*) glycerol (provided as 1X) made up the dilution buffer. The *S. aureus* Topo IV enzyme kDNA (100 ng/L) served as the substrate. STEB: *S. aureus* Topo IV, 40 percent sucrose (*w/v*), kDNA, 10 mM EDTA. Briefly, 100 mM Tris-HCl Bromophenol Blue, 0.5 mg/mL, pH 8, and various concentrations of the investigated substances were combined. For 30 min, the mixtures were incubated at 37 °C. STEB was used to halt the responses. Agarose gel electrophoresis was used to analyze the effects of the process. The gel was then colored with ethidium bromide and photographed under UV light [13,17,43]. 

### 3.7. Docking Studies

The structure of bacterial DNA gyrase (PDB code: 2XCT) was downloaded from the Protein Data Bank. The structure of compound Cpx-Drv was drawn and optimized using the molecular editors MarvinSketch and Avogadro. The protein was prepared using Autodock tools, where the co-crystallized water molecules and Cpx were removed, and Kollman charges and polar hydrogens were then added. The grid coordinates for DNA gyrase were set to 4.800, 44.477, and 67.943 for the x, y, and z axes, respectively, with grid dimensions of 56, 58, and 54. Autodock vina was used for molecular docking and the best docking pose was visualized using Discovery Studio Visualizer [44]. 

### 3.8. Anticancer Activity

MTT assay was implemented to evaluate the cell proliferation of human colorectal carcinoma Hct116 and human liver cancer “HepG2” cell lines, which were implemented at the Science way company and purchased from the ATCC and Asterand. The investigations were conducted as previously mentioned in the study of Al-Hakkani et al. [3,45,46,47,48]. The cytotoxicity profile using the newly prepared Cpx-Drv was demonstrated in the range of concentrations from 1000 to 31.25 μg/mL. A visible spectrophotometer at 560 nm was utilized to test activity.

## 4. Conclusions

A new derivative of Cpx was prepared and identified using various spectroscopic tools. A simple, sensitive, and accurate RP-HPLC method was developed and validated for the concurrent determination of Cpx and Cpx-Drv. Cpx-Drv antibacterial activity was greater against Gram-positive bacteria than against Gram-negative bacteria. Moreover, Cpx-Drv showed remarkable inhibitory activities against DNA gyrase and topoisomerase IV as potential molecular targets. Molecular modeling investigations of Cpx-Drv revealed higher binding interactions for gyrase enzyme (PDB ID: 2XCT) than Cpx, with greater binding affinity.

## Figures and Tables

**Table 1:** FT-IR band assignments of Cpx and Cpx-Drv.

Wavenumber (cm^−1^)		Band Assignments
Cpx-Drv	Cpx	
3316	3389	Stretching vibration band -N-H
3060	3057	Stretching -C=C-H
2951	2922	Stretching aliphatic -C-H of CH_2_ “symmetric”
2811	2832	Stretching aliphatic -C-H of CH_2_ “anti-symmetric”
1728	1720	Stretching vibration -C=O in the carboxylic group
1695	1616	Stretching vibration -C=O of quinoline
1505	1586	Bending vibration -N-H
1361	1375	Hydrated water bending vibration -O-H
1258	1257	Stretching vibration -C-N
1183	1176	Asymmetric -C-O-C
1019	1024	Stretching vibration band -C-F
1004	941	Stretching vibration band C=C bending
826	820	Bending -C=O
806	----	Stretching vibration band -C-Cl
774	785	Bending aromatic -C-H
709	713	Rocking -C-H of >CH_2_
596	567	Aromatic -C-H bending

**Table 2:** System suitability parameters of Cpx.

wt (mg)/200 mL	10.00	10.01	10.02	10.01	10.00	10.01	10.00	10.05	10.51
Replicate #	Optimum	Analyst 2	Day 2	Flow Rate 1.3 mL/min	Flow Rate 1.1 mL/min	ACN % 75%	ACN % 65%	Column-2	Column-3
1	1057.8	1063.2	1060.6	1017.2	1108.3	970.2	1112.8	1080.6	1110.5
2	1058.7	1060.7	1058.3	1017.6	1104.7	971.8	1109.8	1078.3	1107.1
3	1059.2	1060.3	1056.8	1017.0	1105.9	969	1107.7	1079.2	1105.8
4	1058.2	1060.2	1056.9					1079.8	1106.1
5	1060.6	1060.5	1058.1					1093.9	1105.0
6	1060.0	1060.8	1057.4					1094.8	1104.7
Mean	1059.1	1061.0	1058.0	1017	1106	970	1110	1084	1107
STDEV	1.07	1.13	1.40	0.31	1.83	1.40	2.56	7.72	2.12
RSD (%)	0.10	0.11	0.13	0.03	0.17	0.14	0.23	0.71	0.19
USP tailing	1.05	1.09	1.03	1.11	0.82	1.14	0.81	0.76	0.75
Plates	10,086	9906	10,054	10,095	10,081	10,012	10,068	9084	10,016

**Table 3:** System suitability parameters of Cpx-Drv.

wt (mg)/200 mL	10.00	10.01	10.02	10.01	10.00	10.01	10.00	10.05	10.51
Replicate #	Optimum	Analyst 2	Day 2	Flow Rate 1.3 mL/min	Flow Rate 1.1 mL/min	ACN % 75%	ACN % 65%	Column-2	Column-3
1	1294.7	1300.4	1295.6	1241.0	1357.0	1198.1	1413.8	1335.5	1332.7
2	1295.8	1298.1	1293.6	1240.7	1351.4	1195.5	1409.1	1331.3	1327.6
3	1296.4	1297.6	1292.6	1240.9	1353.5	1195.3	1409.4	1331.7	1328.2
4	1296.2	1297.7	1291.7					1332.4	1327.7
5	1296.8	1297.8	1292.6					1337.4	1328.0
6	1295.5	1297.4	1292.1					1337.7	1327.6
Mean	1295.9	1298.2	1293.0	1241	1354	1196	1411	1334	1329
STDEV	0.74	1.12	1.41	0.15	2.83	1.59	2.63	2.90	2.01
RSD (%)	0.06	0.09	0.11	0.01	0.21	0.13	0.19	0.22	0.15
USP tailing	1.13	1.12	1.18	1.15	1.16	1.17	1.15	1.17	1.12
Plates	11,635	11,536	11,439	11,177	11,937	10,882	12,209	11,466	11,451
Resolution	6.995	6.85	6.93	7.04	6.81	7.35	7.25	7.15	7.11

**Table 4:** Cpx and Cpx-Drv linearity data.

Concentration (%)	Concentration (µg/mL)	Cpx Mean P. As	Cpx-Drv Mean P. As
10	5	114.15	136.3
50	25	538.35	666.4
70	35	758.20	939.9
100	50	1066.35	1331.85
120	60	1293.65	1610.85
150	75	1603.50	1993.1
400	200	4235.20	5299.8
Slope		21.12	26.46
Intercept		15.06	9.93
Correlation		0.99999	0.99999
STDEV XY error		7.30	7.21
LOD (µg/mL)		1.14	0.90
LOQ (µg/mL)		3.46	2.72
Accuracy (%) at	35 µg/mL	99.8	100.5
	50 µg/mL	99.8	98.6
	60 µg/mL	100.7	100.2

**Table 5:** Precision, robustness, ruggedness, and specificity results of Cpx and Cpx-Drv.

Item		Cpx	Cpx-Drv
Repeatability as RSD%	Inter-precision	0.51, 0.86	0.79, 0.30
	Intra-precision	0.71	0.57
Robustness as RSD%	Flow rate	0.10, 0.03, 0.17	0.06, 0.01, 0.21
	Organic ratio	0.10, 0.14, 0.23	0.06, 0.13, 0.19
Ruggedness as RSD%	Analyst to Analyst	0.10, 0.11	0.06, 0.09
	Column to Column	0.10, 0.71, 0.19	0.06, 0.22, 0.15
	Day to Day	0.10, 0.13	0.06, 0.11
Specificity as resolution between both Cpx and Cpx Drv	Acid hydrolysis	7.84	
	Base hydrolysis	8.05	
	Light degradation	6.87	

**Table 6:** Antimicrobial activity of Cpx and Cpx-Drv against Gram-positive strains *S. aureus* 6538 and *B. subtilis* 6633, against Gram-negative strains *E. coli* 8739, *P. aeruginosa* 9027, and *B. cepacian* 25416, and against yeast strain *C. albicans*.

Antimicrobial Agent		Inhibition Zone Diameter (mm)				
		Cpx	Cpx-Drv			Fold Increase %
Standard Strains	ATCC	100 ^y^ μg/mL	100 ^x^ μg/mL	50 μg/mL	25 μg/mL	(x − y)/x∗100
Gram-positive strains						
*S. aureus*	6538	6.7 ± 0.3	13.8 ± 0.8	7.0 ± 0.5	0	51.4%
*B. subtilis*	6633	7.8 ± 0.3	11.3 ± 0.6	9.7 ± 0.8	0	30.3%
Gram-negative strains						
*E. coli*	8739	11.2 ± 0.8	15.2 ± 0.3	11.7 ± 0.6	0	26.3%
*P. aeruginosa*	9027	8.7 ± 0.6	13.5 ± 0.9	10.3 ± 0.3	0	35.5%
*B. cepacia*	25,416	0	10.0 ± 0.9	0	0	100%
Yeast strain						
*C. albicans*	10,231	0	0	0	0	---

**Table 7:** Topoisomerase IV and DNA gyrase inhibition assay (IC_50_ in µg/mL) of compounds Cpx and Cpx-Drv.

Item	Topoisomerase IV IC_50_ (µg/mL)	DNA Gyrase IC_50_ (µg/mL)
Cpx-Drv	1.913	6.754
Cpx	0.821	2.125

**Table 8:** Molecular modeling data for both Cpx and Cpx-Drv in the DNA gyrase-active site (PDB: ID 2XCT).

#	Types of Interactions	Ligand Interaction	Binding Affinity
		Amino Acid Residue	(kcal/mol)
Cpx	Metal interaction	Mn^2+^ 2000	−7.7506
	H. bond	Ser 1084	
	Pi−Pi interaction	DG 9	
	Pi−Pi interaction	DT 8	
	Pi−Pi interaction	DC 12	
	Pi−Pi interaction	DA 13	
	Pi−anion interaction	Glu 477	
Cpx-Drv	Metal interaction	Mn^2+^ 2000	−10.3349
	H. bond	Ser 1084	
	Pi−Pi interaction	DG 9	
	Pi−Pi interaction	DT 8	
	Pi−Pi interaction	DC 12	
	Pi−Pi interaction	DA 13	
	Pi−anion interaction	Glu 477	
	Halogen interaction	ILE 461	
	Halogen interaction	LYS 460	

## Data Availability

All data generated or analyzed during this study are included in this article.
